# Sympatric speciation in structureless environments

**DOI:** 10.1186/s12862-016-0617-0

**Published:** 2016-02-29

**Authors:** Wayne M. Getz, Richard Salter, Dana Paige Seidel, Pim van Hooft

**Affiliations:** Department ESPM, University of California, Berkeley, CA 94720-3114 USA; School of Mathematical Sciences, University of KwaZulu-Natal, PB X54001, Durban, 4000 South Africa; Computer Science Department, Oberlin College, Oberlin, OH 44074 USA; Resource Ecology Group, Wageningen University, Droevendaalsesteeg 3a, 6708 PB Wageningen, The Netherlands

**Keywords:** Magic traits, Foraging guilds, Disruptive selection, Genetic algorithms, Agent-based models

## Abstract

**Background:**

Darwin and the architects of the Modern Synthesis found sympatric speciation difficult to explain and suggested it is unlikely to occur. Increasingly, evidence over the past few decades suggest that sympatric speciation can occur under ecological conditions that require at most intraspecific competition for a structured resource. Here we used an individual-based population model with variable foraging strategies to study the evolution of mating behavior among foraging strategy types. Initially, individuals were placed at random on a structureless resource landscape, with subsequent spatial variation induced through foraging activity itself. The fitness of individuals was determined by their biomass at the end of each generational cycle. The model incorporates three diallelic, codominant foraging strategy genes, and one mate-choice or *m*-trait (i.e. incipient magic trait) gene, where the latter is inactive when random mating is assumed.

**Results:**

Under non-random mating, the *m*-trait gene promotes increasing levels of either disassortative or assortative mating when the frequency of *m* respectively increases or decreases from 0.5. Our evolutionary simulations demonstrate that, under initial random mating conditions, an activated *m*-trait gene evolves to promote assortative mating because the system, in trying to fit a multipeak adaptive landscape, causes heterozygous individuals to be less fit than homozygous individuals.

**Conclusion:**

Our results extend our theoretical understanding that sympatric speciation can evolve under nicheless or gradientless resource conditions: i.e. the underlying resource is monomorphic and initially spatially homogeneous. Further the simplicity and generality of our model suggests that sympatric speciation may be more likely than previously thought to occur in mobile, sexually-reproducing organisms.

**Electronic supplementary material:**

The online version of this article (doi:10.1186/s12862-016-0617-0) contains supplementary material, which is available to authorized users.

## Background

Sympatric speciation is thought to be uncommon [1], although several systems—including races of *Rhagoletis pomonella* (apple maggots) and the parasitic Braconid wasps (*Diachasma alloeum*) they host [2], sibling species of *Monostroma* (i.e. *M. latissimum* and *M. nitidummarine*, green algae off the coast of Japan [3]), cichlid species ((*Amphilophus* sp.) complexes in isolated lakes [4] (but see [5]), and the iconic Darwin finches [6]—are considered to be examples of such speciation. The current prevalent view of sympatric speciation is that it is driven by disruptive selection through ecological competition on traits linked to assortative mating mechanisms that, when of genetic origin and not associated with sexual selection, are referred to as “magic traits” [7–11]. The genetic mechanisms underlying sympatric speciation can be quite varied, but they are often thought to involve either some type of recognition system (see [12] and the references therein) or ecological-mediated mate-sorting, such as heteropatry (individuals mate within preferred patch types on a mosaic landscapes [13]). Other, more subtle types of mechanisms also exist [14], suggesting that more mechanisms are likely to be discovered.

Ecological models underlying disruptive selection have invoked either multiple distinct habitat types (which have been referred to as “Levene models” [1]) or a resource gradient, such as seed size in the case of gramnivorous vertebrates. These cases, of course, encompass a considerable variety of situations. An influential, logistic-equation-based, adaptive dynamics analysis by Dieckmann and Doebeli [15] showed that sympatric speciation is a likely outcome of competition for resources. Disruptive selection in their model arose from the competitive exclusion process associated with resource competition processes [16]. In concert with disruptive selection, Kirkpatrick and Ravigne [17] identified a concatenation of mechanisms needed for speciation to occur: an isolating mechanism (e.g. associative mating), a mechanism to link disruptive selection and isolation, a genetic basis for increased isolation, and an appropriate initial situation. Dieckmann and Doebeli’s [15] simulations involved pitting individuals against one another that have different density-dependent growth responses to a given implicit ecological background (the implicitness was reflected in terms of the value of the carrying capacity parameter in the logistic model for the individual in question) and, as such, fell within the growing genre of using individual-based models (IBM) to address ecological and evolutionary questions [18].

Recently, we used an IBM to demonstrated that if a uniform monomorphic resource landscape is peppered at random with consumers that are identical in terms of their ability to compete, extract and convert resources for growth—but employ individually variable movement behavior strategies (in terms of when and where to move, based on evaluations of resource levels and number of competitors in different directions of the compass)—then a polymorphic movement strategy guild emerges with structure dependent on historical quirks [19] rather than on an intrinsic system's attractor. This result considerably weakens the ecological precursors that are necessary for sympatric speciation to occur. Further, if we assume some type of mate labeling cues are linked to behavioral strategies, such as visual coloration correlated with both behavioral type and mating strategies in side-blotched lizard (*Uta stansburiana*) [20] and Midas cichlids (*Amphilophus citrinellus*) [21]—then we have a magic trait system [7–11] that can be used both to promote the reproductive separation of individuals belonging to different behavioral syndromic groups. Further, mating strategies that promote fitness often arise even though we currently may have no verified explanation for their origin, as with observations that olfactory cues are used to avoid sibmating in house mice (*Mus musculus domesticus*) [22]. In our model assortative mating, which is widespread through the animal kingdom [23], promotes fitness, as evidenced by the greater efficiency of clonal versus sexual foraging guilds in exploiting the model resource space [19].

The specific question we address here, in the context of individuals foraging on a structureless resource landscape [19], is the following: if the genetic precursors are in place for a magic-trait system to emerge within a randomly mating population, will assortative mating, as a precursor to sympatric speciation, emerge and become firmly established? The question is answered here in the affirmative, both through our simulation studies and with a well-supported explanation of why we should expect consumer-resource systems to behave in this way. In addition, the results we obtain serve to refute the following hypothesis, articulated in terms of a nicheless resource environment; by which we mean the resource is structurally homogeneous (i.e. it has no spectral qualities such as size or even color variation and exhibits no density gradients, though it can consist of randomly distributed patches or packages of resource):

Hypothesis: *Two closely competing morphs (or strains), coevolving in a mixed population, cannot coexist if they both exploit a nicheless resource environment.*

Given that it only takes one counterexample to disprove an hypothesis, the example we provide ***rejects this hypothesis*** and lays to rest the issue that some kind of resource niche structure is needed to ensure that sympatric speciation can occur when the supporting mechanisms of disruptive selection and genetically driven reproductive isolation, as identified by Kirkpatrick and Ravigne [17], emerge and become fixed. In short, the results we present below demonstrate that different foraging types may not only coexist (as demonstrated in [19]), but that they may be corralled by a magic-trait system [7–11] into mating assortatively, which is a precursor to reproductive isolation and, ultimately, speciation [17].

## Results

### Evolutionary simulations

**Fig. 1:**
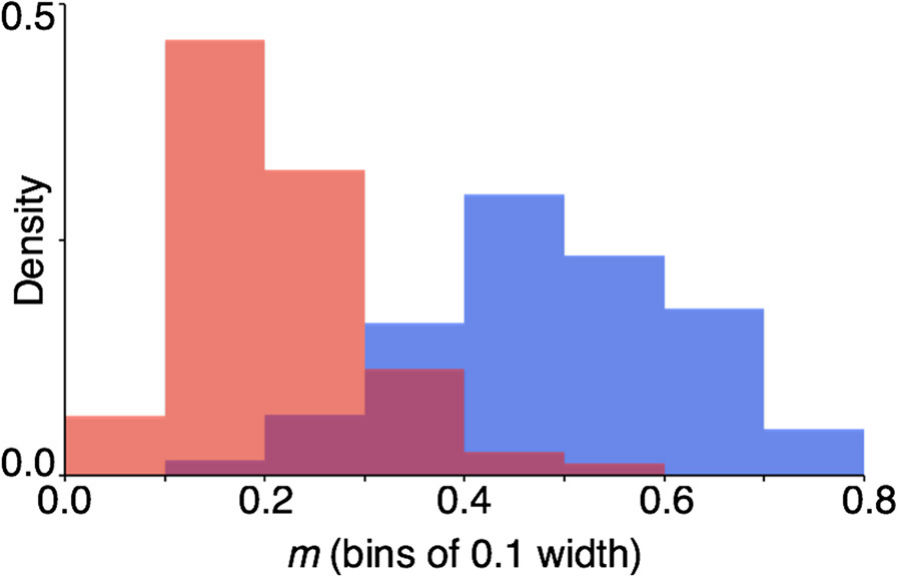
Frequency histograms of the mean value of *m* under random (*blue*) and *m*-trait (*red*) mating (*purple* represents areas of overlap) for the 500 generation cases. See Table 1 for more details

Simulations were carried out using the model described in the Methodology Section, essentially running an evolutionary algorithm on top of our individual-based, single-generation foraging (i.e. ecological) model over evolutionary epochs that were either 250 or 500 generations long. The ranges of final population sizes at generation 250 and 500, together with the means and standard deviations of the mating-phenotype (magic) parameter *m* across runs, as well as mean values of the standard deviation across runs, are given in Table 1. Statistical analyses of these results reveal that the mean values of *m* for the random (*m* = 0.49 ± 0.13, *n* = 124) and *m*-trait (*m* = 0.21 ± 0.09, *n* = 80) mating are significantly different at generation 250, as well as at generation 500 (*n* = 15 for both the random *m* = 0.53 ± 0.20 and *m*-trait *m* = 0.10 ± 0.09 mating cases), suggesting that the *m*-trait gene evolves to promote assortative mating. The distributions of the values of *m* for the 250-generation cases are plotted in Fig. 1, as a histogram binned into 0.1 unit intervals.

**Table 1:** Range of population sizes and basic statistics of the parameter values of *m* for random (value of *m* has no affect) and *m*-trait mating (i.e. assortative mating when *m* < 0.5, dissasortative mating when *m* > 0.5) simulations at the end of the 250 and 500 generation epochs

*N* (runs)	Agent ranges across runs	Mean ± stdev across “within run means”	Mean ± stdev across “within run stdev”
At generation 250^a^			
Random 124	239–361	0.49 ± 0.13^b^	0.17 ± 0.05
*m*-Trait 80	256–348	0.21 ± 0.09^b^	0.11 ± 0.05
At generation 500^c^			
Random 15	265–334	0.53 ± 0.20^b^	0.10 ± 0.07
*m*-Trait 15	263–350	0.10 ± 0.09^b^	0.06 ± 0.04

^a^The difference between distributions of *m* values for random versus *m*-trait mating are plotted in Fig. 1

^b^Significance evaluated using a two-tailed non-parameteric Mann-Witney *U* test. For 250 generations

*p* < <0.001, for 500 generation *p* < 0.01

^c^Additional details in Table 2

### Emergent genetic structure

To provide a sense of the emergent genetic structure at the end of the 500 generation runs (where this structure is more evolved, and hence sharper than after 250 generations), in Table 2 we list the mean values of *m* across all agents in each of the 15 random and 15 *m*-trait mating runs along with the heterozygote deviance (i.e. an index of inbreeding and clustering; see Methods for details) associated with *α*, the most diverse of the three behavioral parameters in the model (See Methods for further explanation). The differences in each of these three measures when compared across random versus *m*-trait mating are all highly significant (*p* < 0.001 in all three cases, Mann–Whitney *U* tests).

**Table 2:** Information on the genetic structure of the population at generation 500 for 15 evolutionary runs under random versus *m*-trait mating (run numbers sorted on *m* separately for cases A. and B.; genetic structure of runs 1 and 15 for both cases illustrated in Fig. 2). The heterozygote deviance value (see Method section) is with respect to the parameter *α*, while the % variance explained by our principal components analysis (PCA) is with respect to the first two components

	A. Random mating^a^			B. *m*-Trait mating^a^		
Run #	*m*	Heteroz. deviance^b^	% PCA Var	*m*	Heteroz. deviance^b^	% PCA Var
1	0.19	0.07	54	0.02	−0.99	80
2	0.31	0.08	53	0.02	−0.97	79
3	0.38	−0.06	54	0.03	−0.98	78
4	0.40	−0.32	58	0.04	−0.99	86
5	0.41	0.03	58	0.04	−0.97	78
6	0.44	0.10	55	0.05	−0.96	78
7	0.45	0.56	55	0.05	−0.96	87
8	0.48	0.04	53	0.06	−0.98	64
9	0.54	−0.14	57	0.06	−0.96	75
10	0.61	NA^c^	55	0.09	−0.95	70
11	0.66	0.10	53	0.11	−0.96	62
12	0.67	0.15	54	0.11	−0.94	60
13	0.76	0.13	57	0.25	−0.84	57
14	0.78	−0.17	57	0.28	−0.83	58
15	0.93	0.19	52	0.28	−0.86	62

^a^Statistical comparisons of A. and B. are highly significant for all three measures using a using a two-tailed non-parameteric Mann-Witney *U* test

^b^A negative value implies a deficit of heterozygotes compared with Hardy-Weinberg equilibrium

^c^No value is given because insufficient genetic structure evolved in the parameter *α* to identify fewer than 5 distinct alleles

**Fig. 2:**
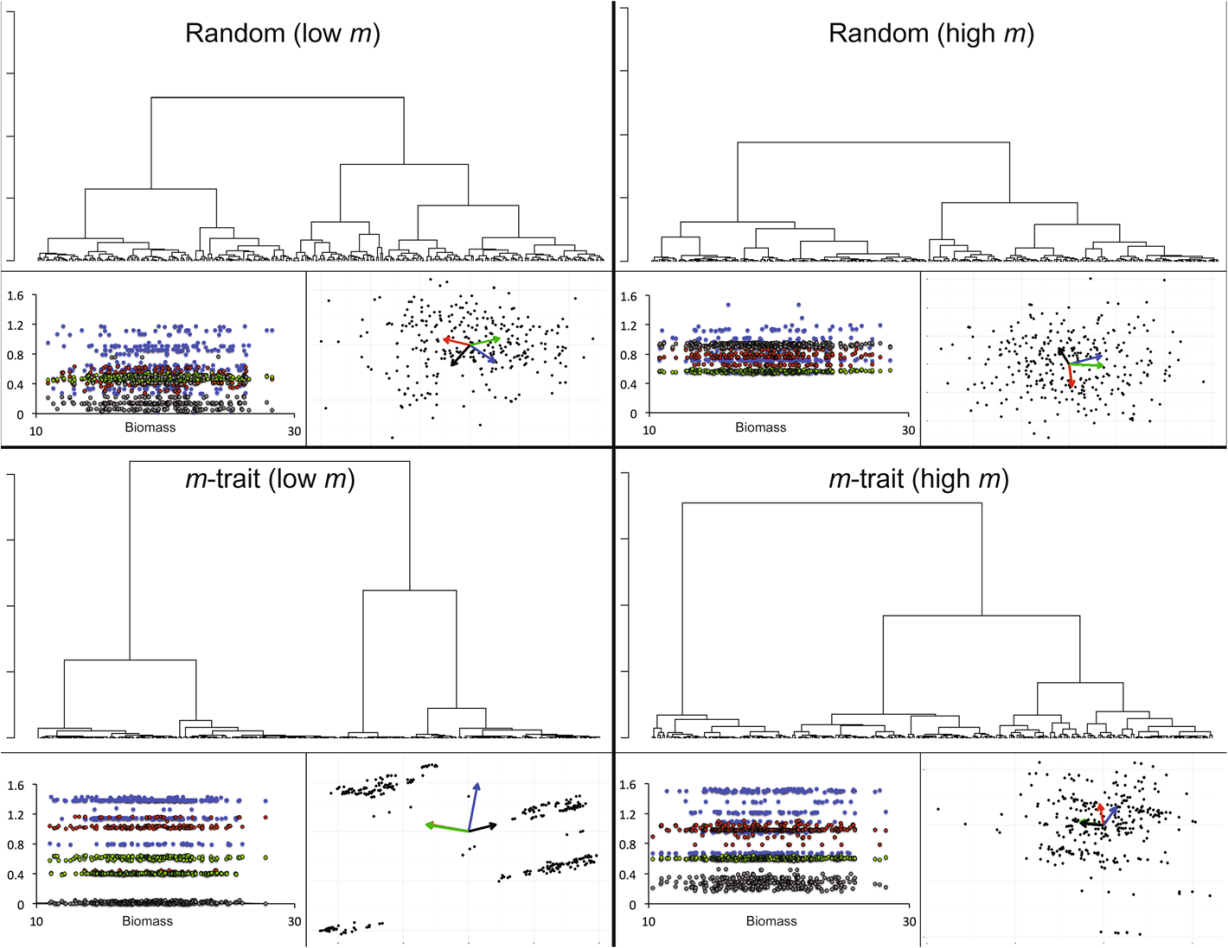
Genetic structure of runs 1 and 15 in Table 2 for the random and *m*-trait mating cases are illustrated here in three different ways (see Additional file 1: Figures S1 and S2 in the supplementary online file for figures depicting all 30 cases for panel types i. and iii.). These are i.) *bottom left panels* for each case: the *α* (*blue*), *δ* (*red*), *ρ* (*green*) and *m* (*black*) parameter values (see Methods Section for an explanation of these parameters) ordered along the horizontal axis according to the final biomass achieved by individuals during generation 500 of the ecological simulation; ii.) *bottom right panels* for each case: a plot of each individual in the space of the first two principal components of a principal components analysis (PCA) for individuals located in the four-dimensional (*α*, *δ*, *ρ*, *m*)-parameter space, with the colored vectors (color coded as in 1. above) indicating the relative weightings of these four parameters (in the *m*-trait cases the weights of two parameters are almost identical resulting in one vector obscuring another); iii) *top panels* for each case: a plot of the dendrogram generated by the PCA

For the four simulations corresponding to the highest and lowest average *m* values for the random and *m*-trait mating cases in Table 2, the genetic structures that emerged at generation 500 are plotted in Fig. 2 (with results from all 30 simulations illustrated in the Additional file 1). Specifically, the plots are: the values of all four parameters (see the Methods Section for an explanation of these parameters) for each individual (Fig. 2, top panels in each of the four cases), the location of each individual in the first two principal components space (Fig. 2, middle panels in each case), and of the dendrograms associated with the principal components analysis (PCA; Fig. 2, bottom panels in each case). The *m*-trait simulations show defined genetic structure has evolved across all four parameters, with the lowest *m* value run of the *m*-trait mating case showing a particularly clear structure (Fig. 2) of two alleles for *δ* (red—the two dominant bands are the homozygote phenotypes), two alleles for *ρ* (green—the two dominant bands are the two homozygote phenotypes), one allele for *m* (black) parameters, and three alleles for *α* (blue—three dominant homogyzote bands plus smaller heterozygote bands). The PCA analysis shows very clear genetic groupings for the case of the smallest *m* value for the *m*-trait mating (Fig. 2) while PCA space grouping are much less distinct for the remaining three cases. Similarly the dendrograms indicate that the populations in the *m*-trait mating cases consist of a couple or several (depending at what vertical axis distance the groups are parsed) groups of more highly related individuals within groups and more distantly related individuals among groups, consistent with the fact that assortative mating groups are forming and increasing their reproductive isolation among groups.

## Discussion

### Interpretation of the results

**Fig. 3:**
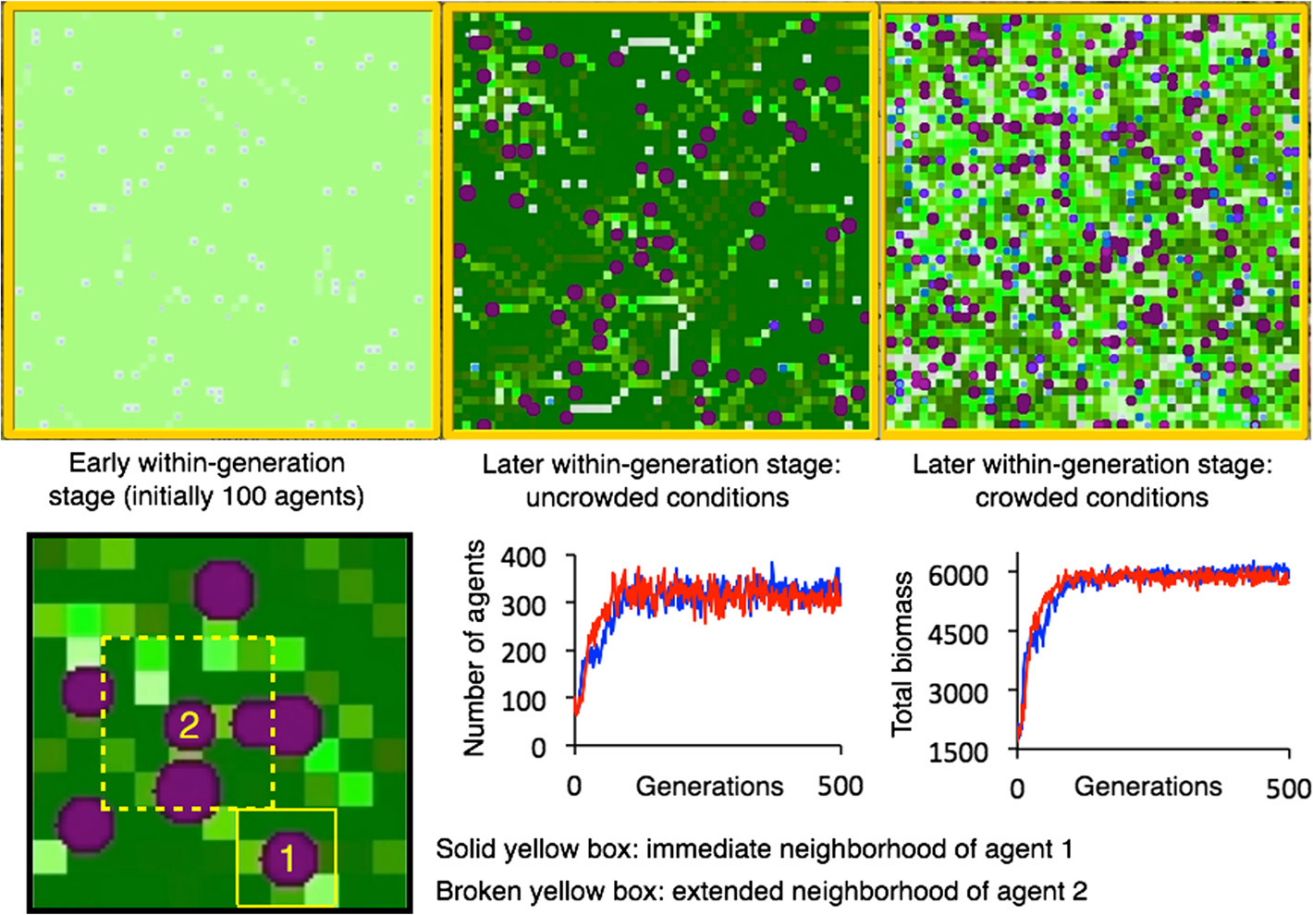
Various stages of the within-generation simulation (*top three panels*) show agent location and biomass state (blue-to-purple circles, size an indication of relative biomass) and within cell resource levels (*light to dark green* indicating low to high resource levels). *Solid yellow rectangle* contains the eight nearest-neighbors around agent 1’s current location); *broken yellow rectangle* contains the eight nearest-neighbors and 16 next-to-nearest neighbors around agent 2’s location. Individuals choose mates from across the whole landscape and not just local neighborhoods. *Red* and *blue* graphs in *middle* and *right bottom panels* show the result from two repeated runs of the number of agents each generation and the total final biomass of these agents and the end of each generation over a 500-generation evolutionary simulation

**Fig. 4:**
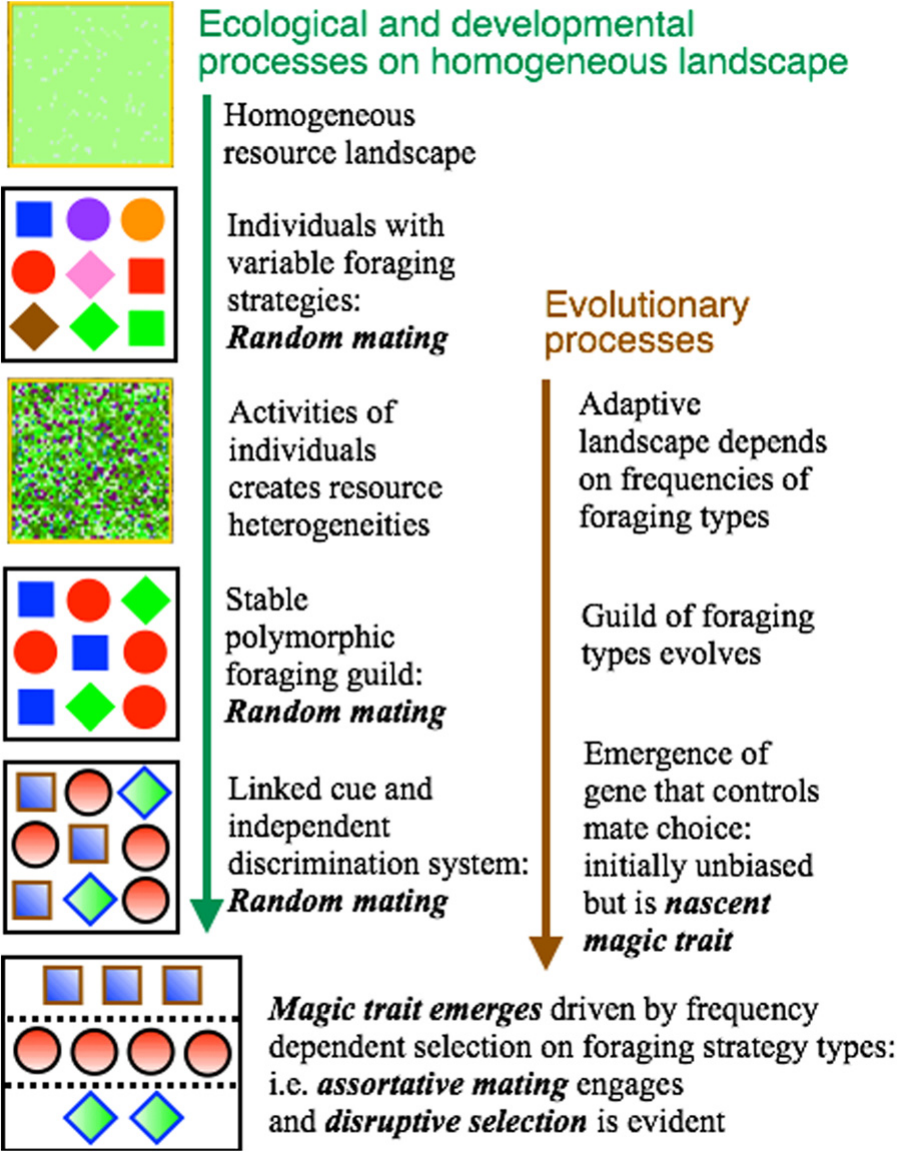
A cartoon of the processes involved in priming the system for sympatric speciation. See text for further discussion

At the start of each generation the resource landscape consists of a uniformly distributed landscape of a monomorphic set of resource patches (top left panel, Fig. 3; top panel Fig. 4), where the resource biomass in each patch changes over time due to resource growth and forager extraction processes [19]. Foragers, in the form of 100 agents are peppered at random over this landscape at the start of each generational cycle. These agents at the start of the evolutionary epoch (250 or 500 generations, as the case may be) have variable foraging strategies assigned to them (second from top panel, Fig. 4). Some time into each intra-generational ecological simulation, the resources on the landscape have increased, except in those cells where agents have been exploiting resources (top middle panel, Fig. 3). Agents with poor foraging strategies appear as small white rectangles, while more successful agents appear as round purple dots in this same panel (bigger dots correspond to agents that have grown the most; cf. top middle panel, Fig. 3).

As time progresses, the number of agents reaches a carrying capacity with stochastic perturbations (bottom middle and right panels in Fig. 3), but the relatively intense level of competition ensures that individuals are smaller on average than we see in the early stages of the evolutionary epoch (low values of the generation index *g*, as *g* increases from 1 to 250 or 500; compare top middle and right panels in Fig. 3), when population levels are around 100 (the first 10 generations) rather than above 300 (>150 generations—see lower middle panel of Fig. 3). In this latter period of intense competition, the homogeneous environment takes on a mosaic structure of resource patches at various stages of regeneration (upper right panel Fig. 3; third panel from the top Fig. 4). At this stage, the optimal foraging strategy type depends on the mix of existing foraging strategy types and is affected by both absolute and relative numbers. Thus, at the start of each generation, the fittest foraging strategy type depends both on the number of within generation foragers and on each of their strategy types. As in the iconic, but deterministic, hawk-dove game the evolutionarily stable strategy (ESS) is a polymorphism represented by a ratio of hawks to doves that satisfies Nash equilibrium conditions (a discussion of the concepts in this sentence can all be found in a review by Nowak and Sigmund [24]). Unlike the hawk-dove game, our system is dynamic with regard to the exploitation of resources by foragers (i.e., during each iteration of the ecological component of the model) and also stochastic. The latter implies that an ESS does not exist in the deterministic sense. To address the question of the existence of a long-run average ESS entails the formulation of the concept of a quasi-stationary strategy (QSS), as defined by Zhou et al. [25]. They demonstrated that a QSS can only be regarded as the long-run average ESS if the stochastic dynamical system’s approach to its QSS is not too rapid. If it is too rapid, as happens in our system, the QSS that emerges is different in each simulation (of 250 or 500 generations in our case). Put in other terms, the dynamic adaptive landscape that is associated with our system’s evolutionary dynamics is the basis for support of a polymorphic guild of foraging strategy types, where the evolving configuration is dependent on the early evolutionary history (i.e. on early stochastic events) of the simulation.

The *m*-trait mating gene that we included in our model, as an extension to our earlier work [19], has the potential to take the population in two directions—assortative or disassortative mating. In our simulation of this extended model we observed that: i) when the gene is inactive (i.e. it does not actually influence mating), the value of *m* wanders in either direction, but across runs realizes an average value very close to *m* = 0.5 (Table 1); ii). when the gene is active (i.e. it influences mate choice in individuals with *m* phenotypes sufficiently different from 0.5: viz. it engages disassortative or assortative mating 72 % of the time when *m* is respectively 0.6 or 0.4: see Methods section for details), the value of *m* evolves downwards over time, as illustrated in the panel second from the bottom in Fig. 4. This evolution of *m* is associated with the emergence of an organized genetic structure (bottom panel in Fig. 4), defined by homozygous individuals grouped into “strain” types. These strains are different behavioral foraging types, with regard to tradeoffs involving avoidance of competition, propensity to move from current to richer resources patches, and one-step-tactical versus two-steps-strategic planning (as described more precisely in the Methods section). Guilds of strains (or foraging types) are better at exploiting adaptive landscapes with multiple peaks (which may dynamically adjust with changes in strain frequencies and environmental factors: e.g., see [26]) than non-guild populations, much as clonal populations better exploit the environment than a randomly reproducing sexual population, as demonstrated in our previous study [19]. Since the integrity of these guilds is eroded by the continual production of heterozygote individuals, evolution drives mate selection to become associative when an *m*-trait system (e.g. recognition based on physical matching of mates to self) that permits mate selection is in place.

### Generality of the model

The model we use is rather generically formulated. Resource consumption is modeled by a Holling type II response function that incorporates interference competition (as originally formulated by Beddington [27] in the context of both predators and parasite search efficiency, by DeAngelis and colleagues [28] in the context of trophic interactions at several levels, and by Getz in the context of biomass transformation webs [29]), but also includes an additional ‘abruptness’ parameter that interpolates between more contest-like and more scramble-like competition [30]. We set the abruptness parameter to be intermediate between these extremes, though interference competition in general for food resources is known to exert a strong selective force on all animals [31]. The movement rules in the model are as applicable to unicellular organisms able to detect resources and conspecifics using chemical gradient signals, as they are invertebrate or vertebrate herbivores, or even omnivores or carnivores if resources patches are suitably scaled to appropriate movement ranges. The primary requirements of the model are that individuals should: i) locally reduce resource density before moving on to areas of greater resource density; ii) have the means to perceive environmental conditions beyond their immediate surroundings (if necessary through the implementation of scouting activities before finally deciding where next to feed); and iii) be able to choose mates based on characteristics correlated with foraging behavior. The model, however, requires no assumptions about the structure of the resource other than the extraordinarily weak assumption that it is locally depletable but renewable. Resources in the model are monomorphic, grow everywhere at the same rate, and are initially homogenously distributed across the landscape. Spatial gradients or distribution across any kind of spectrum (such as seeds occurring in different sizes) are not needed, but any additional resource structure only enhances opportunities for individual specialization to occur, provided this structure is not overly elaborate [32, 33].

### Applicability of the model

Biologists are increasingly identifying species complexes that are best understood in terms of sympatric speciation taking place due to disruptive selection—that is, heterozygotes (hybrids) are less fit than homozygotes (true-species)—and magic trait type mechanisms promoting assortative mating. Merrill et al. [34], for example, have shown in *Heliconius* butterflies that hybrid color-pattern phenotypes are attacked more frequently than parental forms, thereby demonstrating disruptive ecological selection on a trait that also acts as a mating cue. In the side-blotched lizard, *Uta stansburiana*, for example, Corl et al. [20] found geographically widespread throat color polymorphisms where, in some areas, these polymorphisms are reduced in numbers of different morphs. Their phylogenetic reconstructions show that ancestral polymorphisms, though often lost, give rise to morphologically distinct subspecies/species. They further showed that this polymorphism loss was associated with accelerated evolution of sexual dimorphisms, thereby suggesting that polymorphism loss is implicated in species formation. Podos et al. [11], for example, studied the contribution of a magic trait scenario in the divergence of song elements among the Galapagos Santa Cruz Island’s medium ground finches (*Geospiza fortis*). They used the results they obtained to argue that song divergence and discrimination, which are fundamental elements of assortative mating in these finches, is likely fostered in early stages of subspecies divergence under a magic trait mating scenario. Red crossbills provide another ornithological example [35, 36], as likely do some species of ducks [37]; and, in insects, fruitfly provide a possible example [38].

### Foraging behavior and correlated cues

To some extent we should expect foraging behavior to be an expression of a plastic response to environmental cues, though the threshold values of cues for producing behavior could be under genetic control. This is known, for example, to be the case in honeybees [39] (e.g. switching from within hive tasks to foraging). How likely is it, however, that variation in foraging behavior is correlated with detectable visual, auditory or olfactory cues that may be used in an *m*-trait mate selection system? Searle et al. [40] provide a detailed discussion regarding the ubiquity of variation of foraging behavior due to environmental and genetic causes in large mammalian herbivores, with a summary of the mechanisms driving this variation (cf. Table 1 in [40]). Variation can be morphological with associated visual cues: viz. stockier individuals may prevail under contest competition, lither individuals may be more efficient movers, taller individuals more able to assess the state of surrounding resources and so on. Additionally, physiological variation may be correlated with odor cues [41], while morphological variation with auditory cues [42]. In species that have evolved mechanisms to discriminate among individuals—which both invertebrates and vertebrates are able to do using purely genetic cues (i.e. no environmental influences are needed) in contexts as fine as discriminating among individuals based on degree of relatedness to self [43]—these mechanisms are in place to function as an *m*-system, provided mate-selection systems are also in place (which they often are [44, 45]). Additionally, it is conceivable that sympatric species, which vary with respect to foraging behavior, may have separated under a magic trait system that subsequently atrophied (e.g. the cue system disappeared) once other prezygote barriers emerged to entrench speciation.

### Assortative mating systems

In our model, mate selection is not influenced in any way by constraints on movement, because we assume that individuals choose mates from among the population as a whole. In many species, individuals may avoid choosing siblings or even close cousins as mates, with recognition systems evolving to facilitate outbreeding [43]. Beyond this constraint, however, individuals may still assortatively mate, or at least it may be advantageous for them to assortatively mate. For example, it has been shown that individuals in a species of mouse (*Mus spicilegus*) reproduce more rapidly when mates are of similar personality types [46]. Similarly, it has been shown in the great tit (*Parus major*) that parental pairs with similar environmental exploratory rate scores interacted more at the nest than pairs with dissimilar scores [47], while it has been shown in Stellar’s jays that parental compatibility with regard to behavioral type (referred to as behavioral syndromes) increase fitness [48]. While assortative mating purely in the context of genetically expressed phenotype frequencies leads to increased homozygosity, it likely also leads to inbreeding, particularly in small populations, unless a counter prevailing system exists to avoid mating with close relatives. Though we did not include an assessment of inbreeding levels in our *m*-trait mating system, both our random and magic-mating simulations are subject to the same level of 'small population' inbreeding because they involve populations of similar sizes (see “Agent Ranges” column in Table 1). Thus any differences in levels of homozygosity that occur between our two simulation treatments (random vs. magic-mating) cannot be explained in terms of 'small population' inbreeding effects, but are due to assortative mating alone.

### Existence of magic trait systems

How likely is it that magic trait systems exist? Thibert-Plante and Gavrilets [10] used a stochastic, individual-based model to study six different mechanisms of non-random mating, including magic trait mating, evolving in the context of dispersal, niche invasion, and adaptive radiation. As a result of their study, Thibert-Plante and Gavrilets [10] conjecture that mate choice is likely based on a few ‘major traits’ that have direct impact on fitness, which is the case in our model. Further, Thibert-Plante and Gavrilets [10] suggest that magic traits may emerge by co-opting locally adaptive traits for mating decisions, as is also the case in our model. Additionally, Servedio et al. [9] review a variety of mechanisms by which magic traits can be produced and conclude that magic traits occur more frequently than previously thought.

## Conclusion

Our study builds on that of Dieckmann and Doebeli [15] who used an adaptive dynamics approach, in the framework of logistic growth models for a population of phenotypes characterized by a variable *x*, to show that the hypothesis articulated in the opening section of this paper does not hold, thereby rendering speciation a much more likely outcome of competition for resources than previously thought. Implicit in their model is a resource spectrum that implies individuals of phenotype *x* have an environmental carrying capacity *K*(*x*). Additionally we note that this function is an input rather than an emergent property of their model. This model was recently generalized by Haller et al. [33] and used to show that on complex landscapes—which may include environmental gradients, metapopulation structure, and patchiness at different spatial levels of resolution—that intermediate levels of heterogeneity are most likely to lead to the emergence of evolutionary branching, a pattern showed earlier to hold for species as well [32]. Haller et al. [33] also showed that the effects of different types of heterogeneity appear to some extent to be additive in causing evolutionary branching. In another recent study, Debarre [49] asked the question “Can speciation occur in a single population when different types of resources are available, in the absence of any geographical isolation, or any spatial or temporal variation in selection?” and answered the question by stating that “… sympatric ecological speciation is favored when (i) selection is disruptive (i.e. individuals with an intermediate trait are at a local fitness minimum), (ii) resources are differentiated enough and (iii) mating is assortative. In our model, unlike Debarre’s, no resource structure is specified. Further, an implicit resource spectrum is not implied, as in Doebeli and Dieckmann [15] in terms of an input function *K*(*x*), or explicitly specified, as in a recent analysis by Thibert-Plante and Hendry [50]. In fact, we model resource extraction and the ensuing growth effects identically for all individuals: all individuals have precisely the same growth rates and competitive interaction parameter values when exploiting resources at a particular level within any resource cell (patch) on the landscape.

In our model, differences in the strategies of individuals to gather resources over time arises from the particular behavioral strategy employed by individuals to efficiently search out resources over an initially homogeneous resource landscape, while individuals may also move to reduce competitive interactions to differing extents. This initially homogeneous landscape, however, takes on a stochastically generated mosaic structure as a result of the foraging patterns of competitors and of resource regrowth (or replacement) within patches. This induced spatial heterogeneity, without additional gradient structures, is both as simple as can be expected in nature with regard to overall resource structure (the resource is monomorphic with no specified spatial gradients) and more realistic than assuming a constant homogeneous background. The latter follows since all organisms locally deplete resources unless they are located in a constant resource flux (e.g. a spatially homogeneous photon flux and individuals are located so they do not shade the flux from competitors, which is a severely restrictive requirement). Dieckmann and Doebeli [15] also consider the evolution of assortative mating using the type of *m*-gene approach that we took here, and they demonstrated in their model that assortative mating often arises and takes the population in the direction of reproductive isolation among ecologically diverging subpopulations. In our case, though, the divergence emerges from a behavioral polymorphism rather than requiring a structural ecological input through a specified carrying capacity function *K*(*x*). Thus, to the extent that Dieckmann and Doebeli [15] conclude that their “… theory conforms well with mounting empirical evidence for the sympatric origin of many species,” we can conclude the same with less restrictive conditions in that we do not specify any growth rate or other ecologically-related variation with regard to individual phenotypes.

## Methods

### Agent-based consumer-resource model

The model was developed on the Nova Platform [51] following methods more fully described elsewhere [19]. The model is a discrete time, agent-based, stochastic, consumer-resource formulation that simulates the movement behavior and growth of a population of consumers on a cellular array. In each of 100 time steps, representing the passage of a single generation, and a single pass through of the ecological component of the model, individuals can either stay within their current cells (i.e., resource patches) and consume amounts of resources determined by a Beddington-DeAngelis type response function (i.e., compensatory with regard to resource density, decreasing with regard to the level of intraspecific competition and has the same parameter values for all individuals—for more details see [19, 29]), and hence gain in biomass, or individuals can move at some cost to their current biomass to one of 8 neighboring cells (lower left panel, Fig. 3). The resources in cells grow logistically, including a reservoir component (root mass below ground), but lose biomass due to extraction by consumers.

### Foraging strategy within generation simulation

The only variation among individuals is their current location, their accumulated biomass state, and the value of three foraging-strategy parameters: *α*, *δ* and *ρ*. The specifics of how these parameters affect foraging behavior are described below and a description of the mathematical equations used are provided elsewhere [19]. For the sake of clarity, however, we summarize this foraging behavior in terms of the parameter values that represent the (*α*,*δ*,*ρ*)-*strategy phenotypes* of individuals: i) individuals compare the resources and competitors in their current cells, as well as eight neighboring cells, where each cell represents a movement direction, and competitors are counted in terms of the number of individuals that could potentially be inside cells in the next time step; ii) individuals also compute average resource levels and competitors in each of the eight neighboring directions, across those cells that can be reached in two moves (see dotted yellow rectangle in left bottom panel of Fig. 3); iii) individuals weigh the relative value of a unit of resource to the cost of competing with a conspecific, using the parameter value *δ* ≥0 (i.e. *the competition tradeoff parameter*) to obtain a weighted value for each cell; iv) individuals only move to the best neighboring cell if their current cells weighted value is less than *ρ* ≥ 0 times that of the best neighboring cell (i.e. *the movement threshold parameter*); v.) each neighboring cell also includes an average value of that cell’s nearest neighbors discounted by a factor *α* ≥ 0 (i.e. the *next neighbor-discount parameter*). Note that *α* ≥ 1 implies an inflation of the relative importance of the values of the cells two steps removed from an individual’s current location compared with its immediate neighbors. A simulation run of the model begins with an initial number of individuals, which we set to 100 in the first generation (lower middle panel, Fig. 3). For the first few time steps each individual exploits its local patch in an otherwise homogeneous landscape (top left panel, Fig. 3) until individuals with high movement threshold phenotypes values *ρ* move (the closer *ρ* ≥ 0 is to 1 the more readily individuals move). The individuals then accumulate biomass by grazing pathways through a regenerating resource landscape (top middle panel, Fig. 3), with the individual’s biomass state at the end of the 100 times steps (i.e. 1 generation) representing the individual’s relative reproductive fitness.

### Reproduction model

At the end of each 100 step, intergenerational cycle or single pass through of the ecological component of the model, individuals are ranked according to their biomass and then allowed to reproduce sexually, assuming a diploid genetic structure and hermaphroditic mating (i.e. parents pair up, using rules described below, without regard to sexual designation). In a previous study [19], we employed hard selection by allowing the top half of individuals to pair up at random and produce four young each, thereby restoring a preselected number of individuals to compete each generation. Here we employed soft selection as follows. After pairing individuals, we took the average biomass *B*_pair_ of each pair, and produced a pair fecundity index *P*_pair_, based on the maximum biomass *B*_max_ over all individuals: viz., *P*_pair_ = *B*_pair_ /*B*_max._ We then used the binomial distribution with maximum possible number of progeny *n*_max_ to stochastically calculate the actual number of progeny *n*_pair_ ~ BINOMIAL[*n*_max_,*P*_pair_] (for “~” read “is drawn from”). This produced at the end of generation *g* (*g* = 1,…,250 or 500, as the case may be) a total of *N*_*g*+1_ progeny to start off the next generation, each with the same initial biomass condition. Note that each iteration of *g* represents one run through of the ecological component of the model, followed by one run through of the reproduction component of the model. As we see in Fig. 3 (bottom middle panel), for the ecological parameter values used in our model (i.e., those used in [19]), the system evolves from an initial 100 progeny in the first generation to stabilize at an across-generation average of around 300 plus individuals from generation 100 onwards. Note that we used the same parameter values for the ecological component of the model as we did in [19], because the purpose of this study was to not explore the evolutionary aspects of the ecological behavior of the system in more depth, but to extend our previous work to study how easily assortative mating may arise in a systems that has the potential for non-random mating (either assortative or disassortative) to evolve.

### Genetic and magic-trait model

Beyond the three *foraging-strategy phenotype parameters* (*α*,*δ*,*ρ*) included in our previous study [19], we included a fourth *magic trait parameter m*. Because our individuals are diploid, under the assumption that all traits are governed by co-dominant phenotypic determination, individual *k* (*k* = 1,…, *N*_*g*_ in generation *g*) has the following genotype and phenotype:

Genotype of individual *k*: (*α*_*κ*1_, *α*_*κ*2_; *δ*_*κ*1_, *δ*_*κ*2_; *ρ*_*κ*1_, *ρ*_*κ*2_; *m*_*κ*1_, *m*_*κ*1_)

Phenotype of individual *k*: ([*α*_*κ*1_ + *α*_*κ*2_]/2; [*δ*_*κ*1_ + *δ*_*κ*2_]/2; [*ρ*_*κ*1_ + *ρ*_*κ*2_]/2; [*m*_*κ*1_ + *m*_*κ*1_]/2)

In all our simulations, we created from 0 to *n*_max_ (number given by binomial drawing described above) progeny genotypes under Mendelian random segregation: i.e. each parent contributed one of its two alleles at random for each of the parameters in the progeny genotype. We then allowed for mutations, using a procedure described in [19] (i.e. each allele in each progeny could be perturbed by a small amount that declined from around 10 to 0.1 % over time using a simulated annealing approach within our genetic algorithm).

#### Mate choice process

At the start of the evolutionary simulation, the allelic values for *m* of all individuals were all assigned the value 0.5, so non-random mate selection played no role in the mating process. Under random mating, the phenotypic value of *m*, though it would drift, and even self-organize because of linkages that arise to other genes through inbreeding in small population sizes, played no role in mate choice. Under *m*-trait mating, however, the value of *m* played a role in the model as follows.

- The measures *D*_*k*_ = 2*|*m*_*i*_ − 0.5| where calculated thereby insuring 0 ≤ *D*_*k*_ ≤1 for all individuals *k* = 1,…,*N*_*g*_,.
- Starting with the largest individual (ranked by biomass at the end of each generation), individual *k* selected a mate at random with probability 1-(*D*_*k*_)^*γ*^, 0 < *γ* ≤ 1 (thus as *γ* approaches 0 the probability of random mating approaches 0 for all possible value of *D*_*i*_). In our study we used the value *γ* =0.2, which results in non-random mating 72 % of the time when *m* is respectively 0.6 or 0.4.
- We model the assumption that individual *k* could have a sense of its degree of similarity to individual *j*, by defining the measures
  $$ \begin{array}{ccc}\hfill {d}_{kj}=\sqrt{{\left({\alpha}_k-{\alpha}_j\right)}^2+{\left({\delta}_k-{\delta}_j\right)}^2+{\left({\rho}_k-{\rho}_j\right)}^2},\hfill & \hfill j\ne k,\ \hfill & \hfill j=1,\dots, {N}_g\hfill \end{array} $$
  and then applying the following deterministic rules when individuals have been selected to mate non-randomly
  i. If *m*_*k*_ < 0.5 then individual *k* chooses individual *j*, where $j=\underset{i}{ \min}\left\{{d}_{ki}\right\}$
  ii. If *m*_*k*_ > 0.5 then individual *k* chooses individual *j* where $j=\underset{i}{ \max}\left\{{d}_{ki}\right\}$
  iii. If *m*_*k*_ = 0.5 then individual *i* choose individual *j* at random.

Under this algorithm, individuals are increasingly likely to mate non-randomly as their phenotypic value of *m* drifts away from 0.5, mating disassortatively when *m*_*k*_ > 0.5, but mating according to the a magic trait assumption (i.e. assortatively) when *m*_*k*_ < 0.5.

### Analysis of evolutionary data

Initially, we undertook a series of random mating and *m*-trait mating runs of the model over an evolutionary epoch of 250 generations. For the sake of efficiency, we ran these on several different computers simultaneously, experiencing some failures due to web issues and computer crashes. At the point where we had accumulated 124 random mating and 80 *m*-trait mating runs we decided to compare the results using a Mann–Whitney *U* test of the average *m*-trait values across the final population of progeny produced at generation 500. We selected the Mann–Whitney *U* test, rather than the more general Kolmogorov-Smirnov test for differences between two distributions, because we were interested in evaluating shift rather than general shape differences in the two distributions of *m*-trait values. To sharpen the outcomes of the evolutionary process, we conducted an additional 15 runs each of random mating and *m*-trait mate selection of an evolutionary epoch of 500 generations. At the end of each of the runs, we generated a csv (comma separated values) file that organized the following output data, with rows being individuals the following information by columns (phenotype and then genotype information follows the biomass column): Individual#, biomass, *α*, *δ*, *ρ*, *m*, *α*_1_, *α*_2_, *δ*_1_, *δ*_2_, *ρ*_1_, *ρ*_2_, *m*_1_, *m*_2_. We computed the means and standard deviation for each of the columns, as well as the means of these column summary statistics across groups of runs. We also produced graphs of the phenotypes of individuals (vertical axis) organized by biomass of individuals (horizontal axis). We applied cluster analyses (Ward’s method) to the *α*-phenotype data in each run and plotted the resulting phylogenetic trees and the data in the plane spanned by the first two principal components (PC analysis or PCA) of these data. We calculated the heterozygote deviance of the population from Hardy-Weinberg equilibrium [52] using the following formula involving the observed proportion, *H*_obs_, of heterozygotes and the expected proportion, *H*_exp_, at Hardy-Weinberg equilibrium

$$ \mathrm{Heterozygote}\kern0.5em \mathrm{deviance}=\left({H}_{obs}\hbox{--} {H}_{exp}\right)/{H}_{exp} $$

Our reason for focusing on the *α* parameter rather than *δ* or *ρ* (note: an individual may be homogeneous in one and heterogeneous in another of these parameters) is that greatest allelic variation in our model is observed at the *α* gene (i.e. the gene that weighs the relative importance of being tactical versus strategic—i.e. looking at the state of the ‘immediate’ versus ‘next-to-immediate’ neighborhoods).

### Availability of data and materials

This study was not based on empirical data, but rather simulations obtained by running a model built using the Nova Software Platform. This Nova can be downloaded from the Nova Software Website https://www.novamodeler.com/. The software platform is free, but users need to register and obtain a license to run the model under Windows, Mac OS X, and Linux operating systems.

The model itself can downloaded from https://nature.berkeley.edu/getzlab/nova.html by clicking on the link: “Sympatric Speciation Foraging System”.

## Additional file

Additional file 1:
**The supplementary online file contains a link to a related publication, information on running our simulation model using the Nova Software Platform, and Additional file** 1 **: Figures S1 and S2 referred to in the caption to Fig.** 2 **.** (PDF 3328 kb)

## Abbreviations

PCA: principle components analysis
SI: supporting information (file online)
