# Supplementary material for: Sympatric speciation in structureless environments
Source: BMC Evol Biol. 2016 Feb 29;16:50. doi: 10.1186/s12862-016-0617-0 (PMC4770699; doi:10.1186/s12862-016-0617-0)

Supplementary Information.

Links to publication containing additional details on model structure.

Wayne M. Getz, Richard Salter, Andrew J. Lyons, Nicolas Sippl-Swezey, 2015.
Panmictic and Clonal Evolution on a Single Patchy Resource Produces Polymorphic Foraging Guilds (http://journals.plos.org/plosone/article?id=10.1371/journal.pone.0133732)
Research Article | published 14 Aug | PLOS ONE | 10.1371/journal.pone.0133732.

Running the model

The model is run on the Nova Platform that can be downloaded from the Nova Software Website at https://www.novamodeler.com/ . The software platform is free, but users need to register and obtain a license to run the model under Windows, Mac OS X, and Linux operating systems. The models itself can downloaded at http://nature.berkeley.edu/getzlab/nova.html.

Additional Figures S1 and S2.

Figures S1. Genetic structure of runs listed in Table 2 are illustrated here in terms of the parameter values of α (blue), δ (red), ρ (green) and m (black) ordered along the horizontal axis according to the final biomass achieved by individuals during generation 500 of the ecological simulation. **Panels A**: random cases. **Panels B**: m-trait mating cases.

Fig. S1: Panels A.

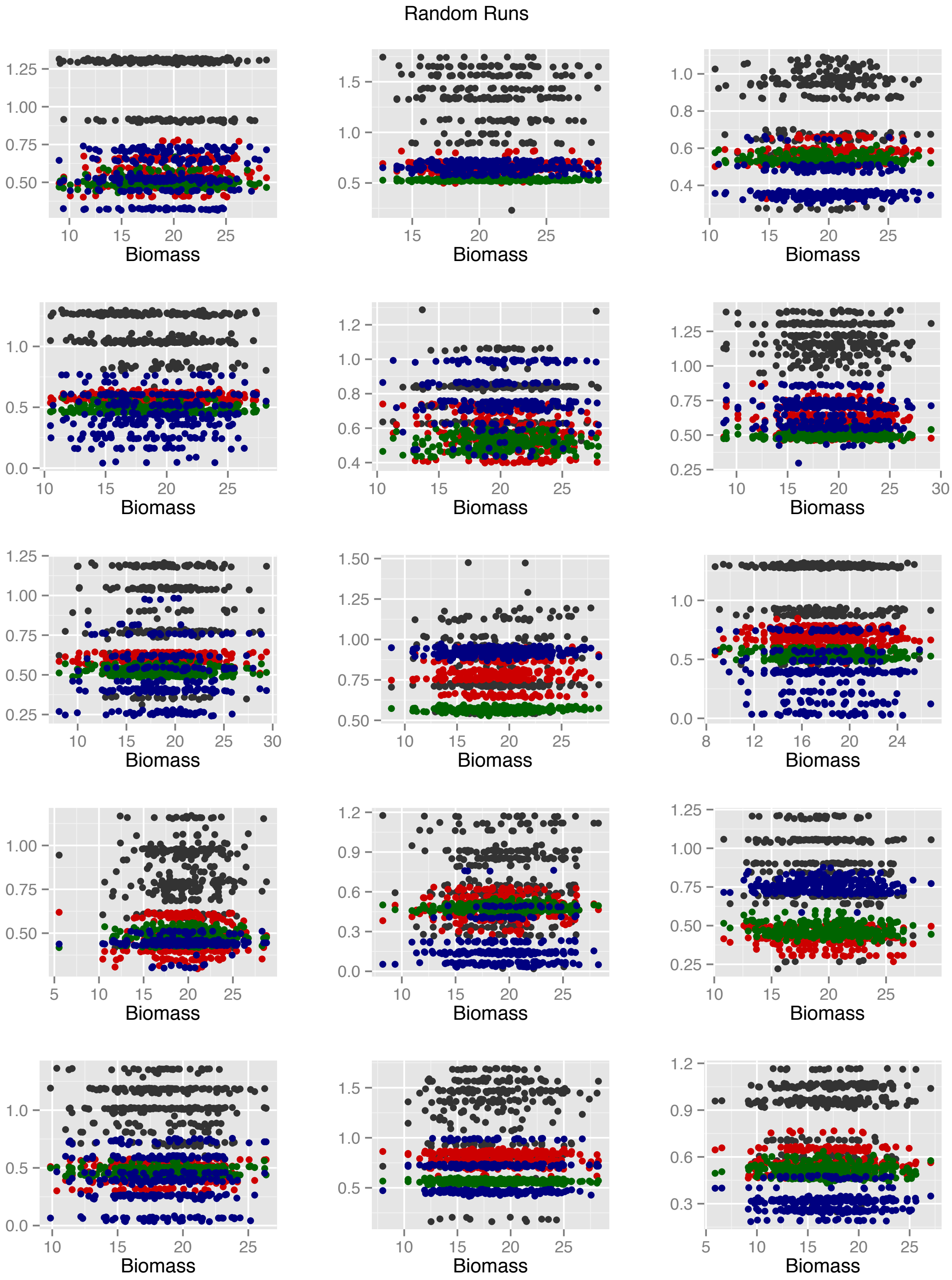

Fig. S1: Panels B.

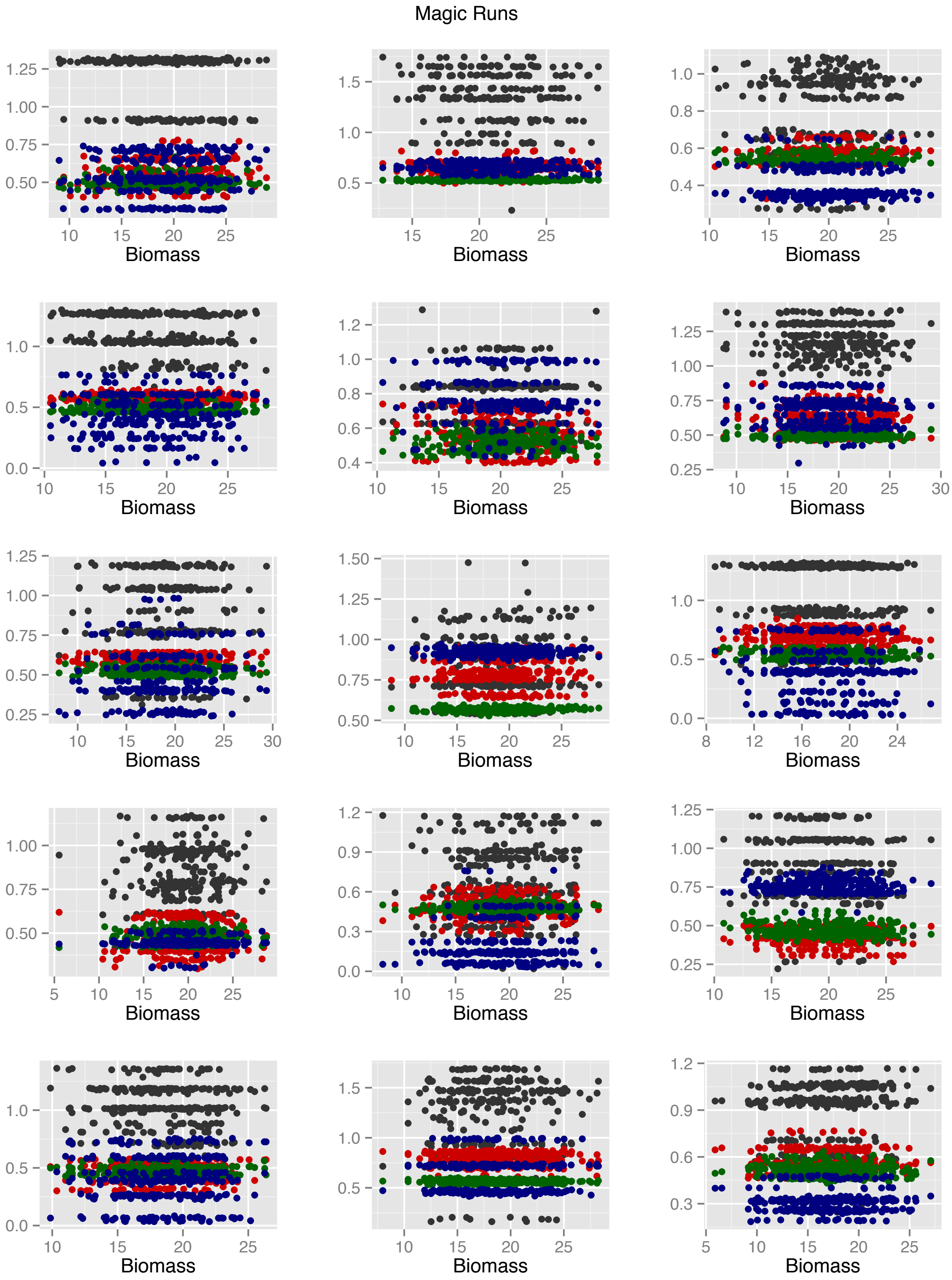

Figures S2. Dendrograms associated with the genetic structure of runs listed in Table 2 are illustrated for all 15 random (**Panels A**) and *m*-trait mating (**Panels B**) cases.

Fig. S2: Panels A.

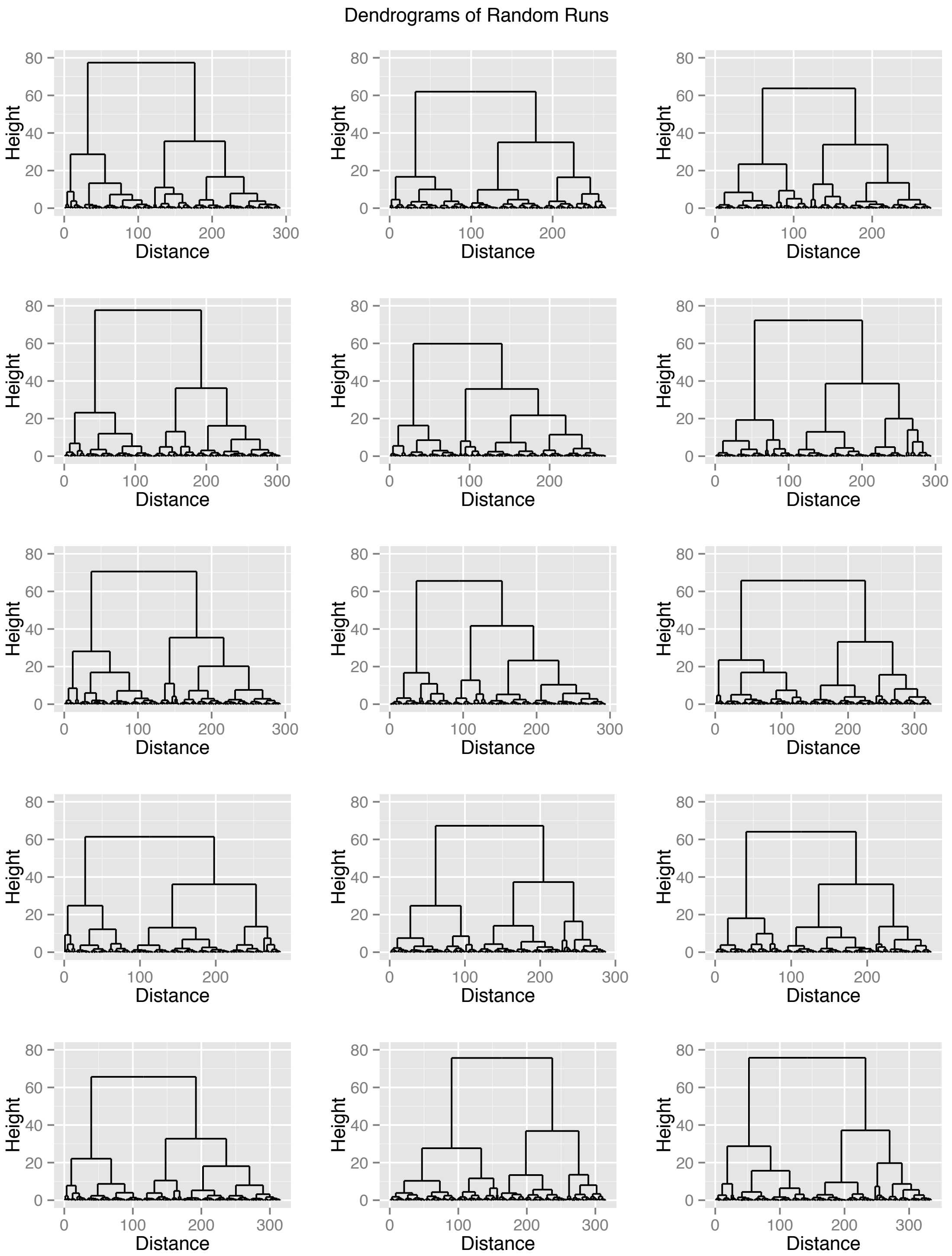

Fig. S2: Panels B.

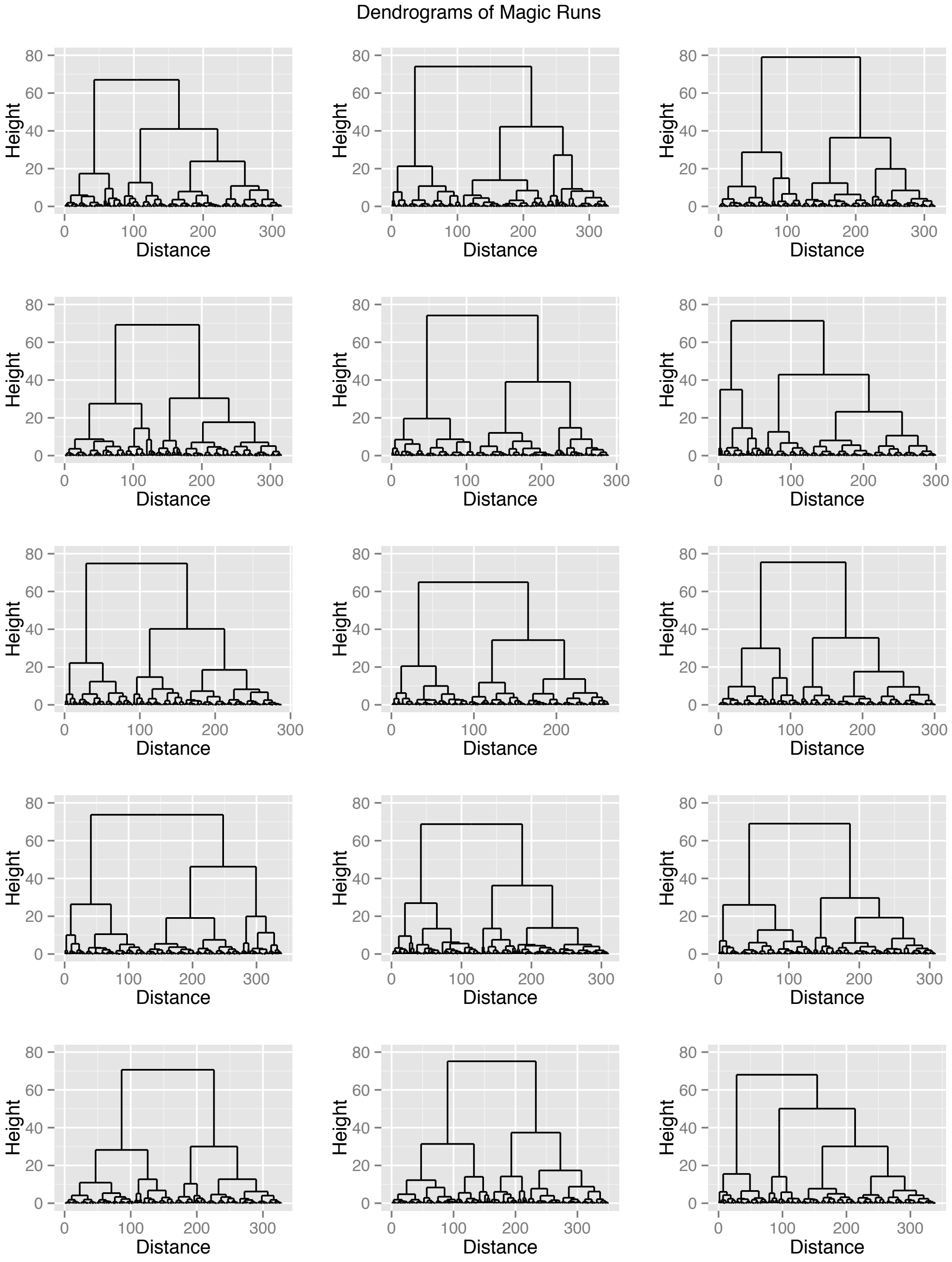

Supplement: Additional file 1: — The supplementary online file contains a link to a related publication, information on running our simulation model using the Nova Software Platform, and Additional file 1 : Figures S1 and S2 referred to in the caption to Fig. 2 . (PDF 3328 kb) [file 12862_2016_617_MOESM1_ESM.pdf]
